# Pentalogy of Cantrell Marked With Ectopia Cordis, Gastroschisis, and Cystic Hygroma in the First Trimester: A Rare Case

**DOI:** 10.7759/cureus.69360

**Published:** 2024-09-13

**Authors:** Jonathon H Boyd, Sean Lief, Ali Z Ansari, Axel B Lichtenberg, Michael De La Paz, Srihita Patibandla, Samuel F Brown

**Affiliations:** 1 Department of Obstetrics and Gynecology, William Carey University College of Osteopathic Medicine, Hattiesburg, USA; 2 Department of Internal Medicine, William Carey University College of Osteopathic Medicine, Hattiesburg, USA; 3 Department of Pathology and Laboratory Medicine, William Carey University College of Osteopathic Medicine, Hattiesburg, USA; 4 Department of Orthopedic Surgery, William Carey University College of Osteopathic Medicine, Hattiesburg, USA; 5 Department of Internal Medicine, Trinity Health Grand Rapids, Grand Rapids, USA; 6 Department of Obstetrics and Gynecology, South Central Regional Medical Center, Laurel, USA

**Keywords:** cystic hygroma, ectopia cordis, extreme obesity, first-trimester, gastroschisis, high-risk pregnancy, hypothyroidism, maternal and fetal medicine, obstetrics and gynecology, threatened abortion

## Abstract

Pentalogy of Cantrell is a rare congenital syndrome characterized by defects in the abdominal wall, sternum, diaphragm, and heart. A severe manifestation of this syndrome is ectopia cordis, where the heart is located partially or entirely outside the chest cavity. Gastroschisis involves a defect in the abdominal wall, where the intestines protrude outside the abdomen without a protective membrane. Cystic hygroma is a malformation of the lymphatic system leading to fluid-filled cysts. We present the case of a 29-year-old G3P0020 female initially seen for obstetric follow-up due to a threatened abortion at 11 weeks of gestation with vaginal bleeding. During a routine limited outpatient ultrasound at 11 weeks and four days of gestation, the heart was found to be entirely extrathoracic. Further ultrasound imaging confirmed the presence of ectopia cordis, gastroschisis, and cystic hygroma. Despite the poor prognosis and high risk of intrauterine fetal demise, the patient was referred to a higher-level care facility. This case highlights the critical role of first-trimester ultrasound in diagnosing severe fetal anomalies and the importance of early recognition and prompt referral for the best possible outcomes.

## Introduction

Ectopia cordis is an exceptionally rare congenital anomaly characterized by the partial or complete displacement of the heart outside the thoracic cavity. Its estimated annual incidence ranges from 5.5 to 7.9 per million live births, with detection possible as early as the first trimester using transabdominal ultrasound [1]. Some studies suggest that first-trimester ultrasound has a sensitivity as high as 93.26% for detecting ectopia cordis [2]. This condition is often associated with pentalogy of Cantrell, a syndrome involving five congenital defects affecting the sternum, pericardium, diaphragm, anterior abdominal wall, and heart [3]. The prognosis for ectopia cordis is generally poor. In a study of 31 cases, 15 pregnancies were terminated, seven resulted in spontaneous fetal demise, and only seven fetuses survived to birth. Notably, of those who survived, all three who underwent surgical correction survived [1]. This emphasizes the critical role of surgical intervention in the management of ectopia cordis.

Gastroschisis is a congenital defect of the abdominal wall where the bowel protrudes through the defect without a protective membrane. Infants with gastroschisis, of whom up to 80% are born prematurely with an average gestational age of 36 weeks, are at high risk for growth failure, with 15% also experiencing intrauterine growth restriction [4]. Notably, like ectopia cordis, gastroschisis has been described as one of the potential defects in pentalogy of Cantrell [5]. The condition is often correlated with elevated maternal levels of alpha-fetoprotein. Prompt surgical intervention after birth can result in a favorable survival rate for neonates with gastroschisis [6]. Infants born with this condition tend to have better outcomes when delivered at facilities equipped with pediatric surgeons, high-risk obstetricians, and neonatologists, ensuring comprehensive care from an advanced interprofessional team [4].

Cystic hygroma is a vascular anomaly characterized by malformation of the lymphatic structures, most commonly occurring in the neck. When detected in utero, the prognosis is particularly poor, especially when accompanied by other abnormalities, such as cardiac defects. Cystic hygroma is strongly associated with aneuploidies, including Trisomy 13 (Patau syndrome), Trisomy 18 (Edwards syndrome), and Trisomy 21 (Down syndrome) [7]. In our review of the literature, we found no reports of a first-trimester fetus presenting with the simultaneous occurrence of all three anomalies: ectopia cordis, gastroschisis, and cystic hygroma.

## Case presentation

**Figure 1:**
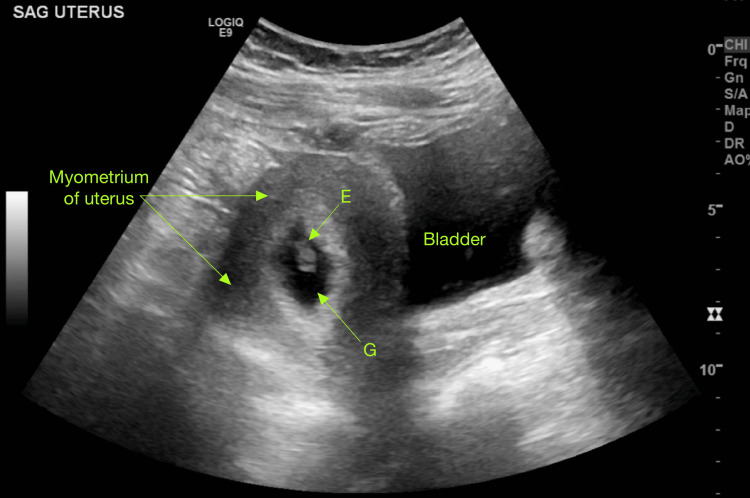
Sagittal view of the uterus at seven weeks and three days of gestation, demonstrating a viable intrauterine pregnancy E: embryo, G: gestational sac

A 29-year-old African American female, gravida 3 para 0-0-2-0 (G3P0020), presented at 11 weeks and four days of gestation for a routine obstetric visit and limited ultrasound. Her medical history includes hypothyroidism, two prior spontaneous abortions, a BMI over 50, and insomnia. She has had two previous dilatation and curettage procedures. Her family history is significant for diabetes mellitus in both her mother and sister. The patient is currently taking prenatal vitamins and melatonin. She reported that all three pregnancies were with the same partner. One month prior to this visit, the patient was diagnosed with cystitis and trichomoniasis after presenting to the emergency department (ED) with dysuria. A positive urine beta-human chorionic gonadotropin (β-HCG) test confirmed the pregnancy, and she was prescribed metronidazole (Flagyl) and cephalexin (Keflex). Urinalysis revealed urobilinogen, blood, ketones, protein, nitrites, leukocyte esterase, epithelial cells, white blood cells, red blood cells, bacteria, and trichomonas (Table 1). Based on her last menstrual period, she was estimated to be at seven weeks and one day of gestation at that time. She followed up with her obstetrician/gynecologist (Ob/Gyn) a couple of days later to establish prenatal care, where an ultrasound confirmed an intrauterine pregnancy without congenital anomalies (Figure 1). Three days following this visit, she experienced vaginal bleeding and was diagnosed with a threatened abortion. On follow-up with her Ob/Gyn, she was found to have a closed cervix, a viable intrauterine pregnancy, and resolved bleeding, with no further complaints.

**Table 1:** Results of the urinalysis during initial visit at the ED HCG: human chorionic gonadotropin, ED: emergency department, HPF: high power field

Components of urinalysis	Value	Reference range
Color	Yellow	Yellow
Clarity	Hazy	Clear
Specific gravity	≥1.030	1.000-1.060
pH	6.0	5.0-9.0
Urobilinogen	1.0 mg/dL	0.1-1.8 mg/dL
Blood	Large	Negative
Glucose	0	0-15 mg/dL
Ketones	40 mg/dL	<1 mg/dL
Protein	100 mg/dL	<10 mg/dL
Nitrite	Positive	Negative
Leukocyte esterase	Positive	Negative
Epithelial cells	Too many to count	<15 cells per HPF
White blood cells	Too many to count	<5 cells per HPF
Red blood cells	Too many to count	<4 cells per HPF
Bacteria	>100,000 colony forming units	<100,000 colony forming units
β-HCG	Positive	Negative

Prenatal screening, including non-invasive prenatal testing (NIPT), showed no abnormalities (Table 2), and the patient's thyroid-stimulating hormone level was 1.14, indicating well-controlled hypothyroidism. At 11 weeks and four days of gestation, a transvaginal ultrasound suggested the presence of severe congenital abnormalities, leading to a referral to a local hospital for further radiological evaluation and an official radiology report.

**Table 2:** Results of prenatal screening, including NIPT NIPT: non-invasive prenatal testing

Test	Result	Reference range
Down syndrome	Not detected	Not detected
Edwards syndrome	Not detected	Not detected
Patau syndrome	Not detected	Not detected
Turner syndrome	Not detected	Not detected
Jacobs syndrome	Not detected	Not detected
Klinefelter syndrome	Not detected	Not detected
Triple X syndrome	Not detected	Not detected
22q11 deletion (DiGeorge syndrome)	Not detected	Not detected
15q11 deletion (Angelman syndrome and Prader-Willi syndrome)	Not detected	Not detected
11q23 deletion (Jacobsen syndrome)	Not detected	Not detected
8q24 deletion (Langer-Giedlion syndrome)	Not detected	Not detected
5p15 deletion (Cri-du-chat syndrome)	Not detected	Not detected
4p16 deletion (Wolf-Hirschhorn syndrome)	Not detected	Not detected
1p36 deletion syndrome	Not detected	Not detected
Trisomy 16	Not detected	Not detected
Trisomy 22	Not detected	Not detected

**Figure 2:**
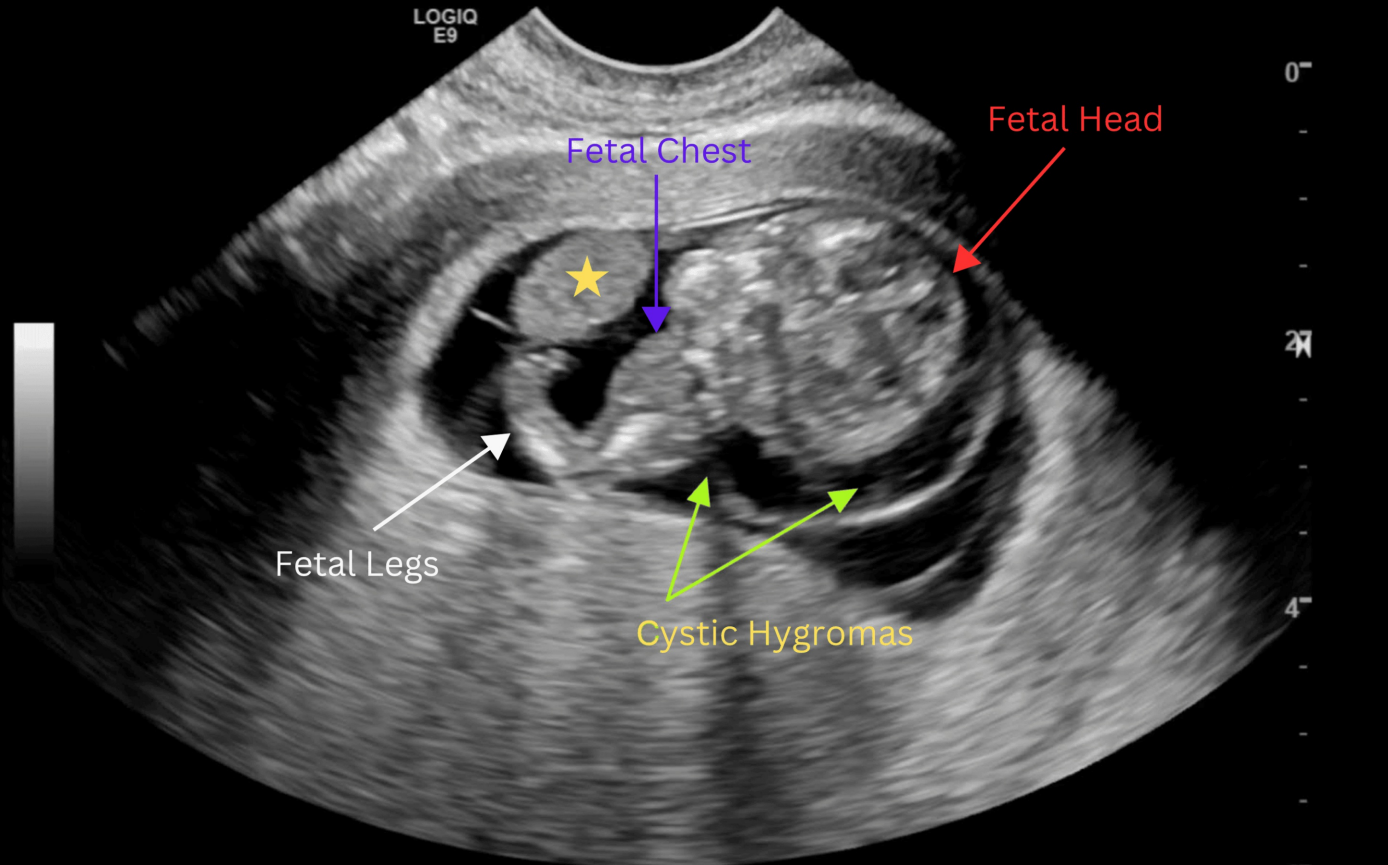
Sagittal ultrasound of the uterus at 11 weeks and four days of gestation, demonstrating findings suggestive of cystic hygroma (yellow arrows) and partial liver herniation (yellow star). Additional landmarks for orientation include the fetal head (red arrow), fetal chest (indigo arrow), and fetal legs (white arrow)

**Figure 3:**
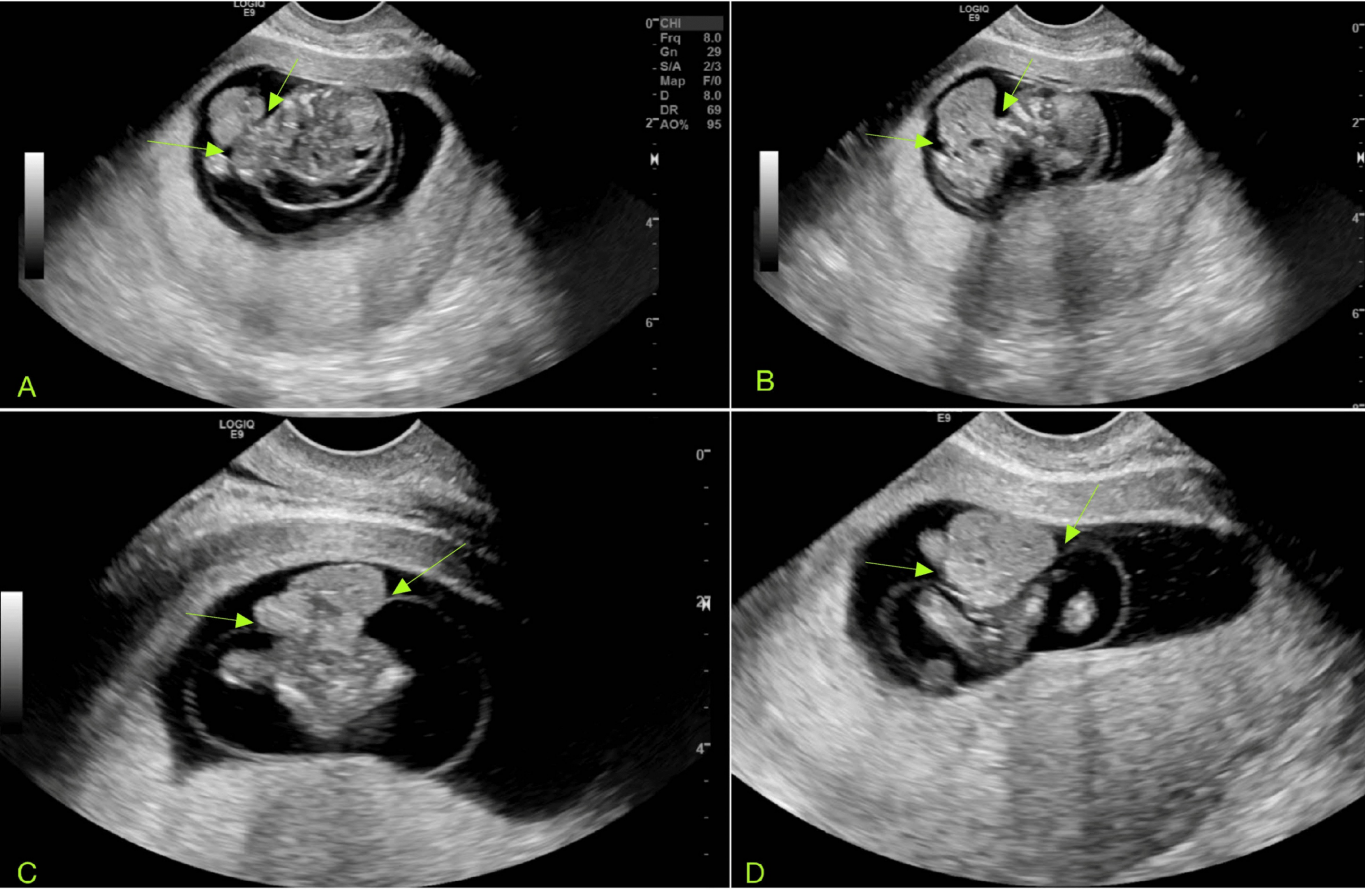
Images A and B display sagittal ultrasound views of the ventral defect (yellow arrows), while Images C and D present transverse views of the same defect (yellow arrows), raising concern for gastroschisis

**Figure 4:**
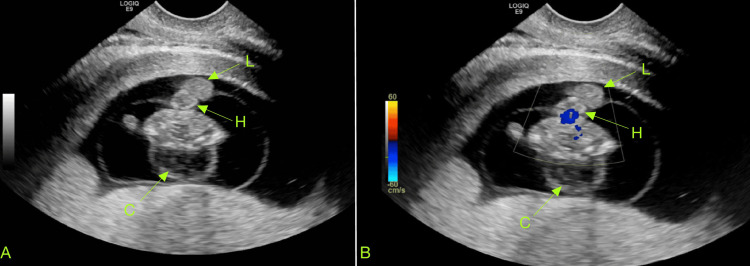
Transverse ultrasound view showing the fetal heart and part of the herniated liver outside the fetus. Image A provides a standard view for comparison, while image B is a Doppler ultrasound demonstrating blood flow through the heart C: cystic hygroma, H: heart, L: liver

Ultrasound determined the fetal heart rate to be 174 beats per minute (bpm). An abnormal appearance of the fetus was noted, including a hypoechoic region along the fetal head and neck, raising concern for cystic hygroma (Figure 2). Additionally, echogenic material was observed outside the fetus, likely representing fetal viscera, which suggested a developmental anomaly with a body wall defect, such as gastroschisis (Figure 3). Furthermore, the fetal heart also appeared to be positioned externally (Figure 4 and Video 1).

**Video 1:** Visualization of ectopia cordis with the fetal heartbeat evident

At 13 weeks, a follow-up transvaginal ultrasound detected a fetal heart rate of 157 bpm. The patient was then informed about the presence of multiple severe congenital anomalies and was advised of the high likelihood of fetal demise. She was referred to a maternal-fetal medicine specialist and a highly equipped multidisciplinary care center for a comprehensive evaluation and management.

## Discussion

Ectopia cordis can be classified into four subtypes based on the anatomical location of the heart: thoracic, thoracic-abdominal, abdominal, and cervical. The thoracic type, in which the heart protrudes outside the thoracic cavity without any overlying structures, is the most common. The thoracic-abdominal type, characterized by the heart's protrusion involving both the thoracic and abdominal cavities, is most frequently associated with the rare congenital anomaly known as pentalogy of Cantrell [8,9]. Our case aligns with this thoracic-abdominal type. The exact etiology of ectopia cordis remains unclear, but it is believed to involve abnormalities in ventral folding morphogenesis, a process crucial for the development of the heart tube and closure of the ventral body wall. Various theories have been proposed, including the arrest of descent of the lateral body folds, rupture of the chorion leading to failure of midline fusion, and the involvement of amniotic bands, which together could result in the severe malformations characteristic of Cantrell's syndrome, such as the heart protruding outside the chest. Recent studies have suggested a link between alterations in bone morphogenic protein 2 (BMP-2) expression and ventral wall defects that can lead to ectopia cordis [8]. In our case, the patient presented with a threatened abortion during the early stages of pregnancy, which may have contributed to this developmental defect. The potential insult during this critical period could have disrupted normal embryological processes, leading to the malformation.

A study examining outcomes for ectopia cordis found that 66% of the cohort were classified as very-low-birth-weight infants. Among these infants, only 6% either survived to discharge or were still alive after one year of hospitalization and inpatient treatment. In contrast, 41% of non-very-low-birth-weight infants in the same patient population met the same survival criteria [10]. These findings suggest that while the overall prognosis for ectopia cordis remains poor, ensuring adequate nutrition in utero to prevent very-low-birth-weight infants might improve outcomes. Future research could focus on strategies to optimize prenatal nutrition and care to promote better survival rates for these infants. For more detailed evaluation following a routine obstetric ultrasound, clinicians could consider fetal echocardiograms to assess for additional congenital heart diseases, such as tetralogy of Fallot, truncus arteriosus, double-outlet right ventricle, or ventricular septal defects. Unfortunately, in our case, a fetal echocardiogram was not available due to local resource limitations [1].

The prognosis and outcomes for gastroschisis are complex and highly variable, depending on whether it is an isolated anomaly or accompanied by other anomalies. Infants with additional anomalies, particularly congenital cardiac anomalies like our patient, often have significantly worse outcomes and account for the majority of in utero fetal demises [11]. Compared to neonates born without abdominal wall defects, those with gastroschisis face a poor prognosis when complications such as sepsis, necrotizing enterocolitis, or short bowel syndrome occur [11,12]. A literature review highlighted that in studies analyzed, 80% of infants with gastroschisis underwent immediate surgery. Of these, 69% had primary closure performed by manually returning the bowel to the abdominal cavity, while 31% underwent repair using a sterile silo-based method [12]. The risk factors for the development of gastroschisis are not well understood, but there is evidence of a correlation with genitourinary infections acquired before or during the first trimester. This aligns with our patient's case, as they tested positive for cystitis-induced trichomoniasis on the day the pregnancy was first identified [6].

Cystic hygroma, also known as nuchal edema, most commonly occurs in the neck region, accounting for 75% of cases, as observed in our patient. The axilla is the second most common site, representing 20% of cases [7]. About half of cystic hygromas are associated with aneuploidies, including Turner syndrome and Trisomy 21, the latter being the most frequently associated. There is also a known correlation with DiGeorge syndrome (22q11 deletion) [7]. Cystic hygroma has been identified in other cases presenting simultaneously with pentalogy of Cantrell. For instance, a case report described twin fetuses both presenting with cystic hygroma and congenital defects associated with Cantrell’s syndrome, detected via ultrasound. In that case, the defects were first identified at 10 weeks and five days of gestation, whereas in our case, anomalies were recognized at 12 weeks and one day of gestation [13]. When cystic hygroma is the sole anomaly and the karyotype is normal, the size of the hygroma can be a useful prognostic indicator. A nuchal size greater than 6.5 mm is associated with a poorer prognosis, while a size less than 6 mm is associated with a more favorable prognosis [7]. However, in our case, we did not use this measurement due to the presence of other anomalies that precluded an isolated prognostic assessment, along with the possibility of spontaneous resolution of cystic hygroma in utero [14].

In cases of suspected pentalogy of Cantrell, the first indication may come from a routine ultrasound performed in the late first trimester [1]. Clinicians should be aware that pentalogy of Cantrell can be classified into three categories: Class 1 is a definitive diagnosis involving all five defects, Class 2 is a probable diagnosis with at least four defects, and Class 3 represents an incomplete expression with some of the defects present [9]. Due to resource limitations, our case falls under a Class 3 categorization. However, when available, fetal echocardiography or fetal MRI can be used to further characterize defects prenatally and assess their severity [1,9,15]. If the fetus survives, further evaluation of the defects can be conducted in the postnatal period using various modalities, including chest X-rays, echocardiography, cardiac catheterization, CT angiography, and surgical assessment [15]. When an anomaly is detected on prenatal ultrasound, we recommend non-invasive prenatal testing, which has high sensitivity and specificity for detecting common aneuploidies and chromosomal deletions through the analysis of cell-free fetal DNA in maternal circulation [16]. Clinicians should also consider that pentalogy of Cantrell and cystic hygroma are strongly associated with Trisomy 21, Trisomy 18, and Turner syndrome, each of which carries significant prognostic implications [7,16]. Importantly, if a fetus with ectopia cordis survives to birth, birth weight is a key prognostic factor [10]. Given the complex nature of this condition, delivery should be planned at a well-equipped facility with a multidisciplinary team specialized in handling such cases, as surgery is crucial for survival. As soon as pentalogy of Cantrell is suspected, prompt referral to a comprehensive care center is essential [1,12,15].

## Conclusions

This case highlights the rare occurrence of pentalogy of Cantrell, particularly through the detection of ectopia cordis, cystic hygroma, and gastroschisis, representing a novel presentation of the syndrome. It emphasizes the critical role of routine prenatal ultrasound in detecting anatomical anomalies, which should be followed by a thorough evaluation by obstetricians. Early ultrasound and appropriate prenatal screenings can identify pentalogy of Cantrell as early as the first trimester, especially in cases with severe malformations. Clinicians should recognize that pentalogy of Cantrell can present with a broad spectrum of defects, beyond those observed in this case. The complexity of pentalogy of Cantrell necessitates a coordinated, multidisciplinary approach to evaluation and treatment planning. Prenatal planning should include surgical consultation, as timely surgical intervention remains a crucial option for infants born with pentalogy of Cantrell, particularly in cases involving ectopia cordis. Future research should focus on identifying maternal risk factors contributing to the development of pentalogy of Cantrell and exploring innovative strategies to prevent in-utero and postnatal fetal demise.
